# Reliability, Concurrent Validity, and Clinical Performances of the Shorter Version of the Roland Morris Disability Questionnaire in a Sample of Italian People with Non-Specific Low Back Pain

**DOI:** 10.3390/jpm14070740

**Published:** 2024-07-11

**Authors:** Teresa Paolucci, Letizia Pezzi, Daniele Coraci, Lucrezia Tognolo, Andrea Pantalone, Carmine Attanasi, Giancarlo Graziani, Davide Dalla Costa, Federico Arippa, Alice Cichelli, Marco Monticone

**Affiliations:** 1 Department of Oral, Medical and Biotechnological Sciences (DSMOB), Physical Medicine and Rehabilitation, University of Study “G. D’Annunzio” of Chieti, 66100 Chieti, Italy; teresa.paolucci@unich.it (T.P.); alice.cichelli@unich.it (A.C.); 2 Rehabilitation Unit, ASST Cremona-Ospedale di Cremona, Viale Concordia, 1, 26100 Cremona, Italy; 3 Department of Neuroscience, Section of Rehabilitation, University of Padova, 35122 Padua, Italy; daniele.coraci@unipd.it (D.C.); lucrezia.tognolo@unipd.it (L.T.); 4 Department of Medicine and Science of Aging, University “G. D’Annunzio”, 66100 Chieti, Italy; andrea.pantalone@unich.it; 5 Santa Caterina Novella Hospital, 73013 Lecce, Italy; carmine.attanasi@asl.lecce.it; 6 Dipartimento Specialita’ Mediche, Uoc Medicina Riabilitativa, Asl Roma 1, Borgo Santo Spirito, 3, 00193 Rome, Italy; giancarlo.graziani@aslroma1.it; 7 Unit of Rehabilitation Medicine and Neurorehabilitation, Department of Neuroscience, ASST Niguarda Hospital, 20162 Milan, Italy; davide.dallacosta@ospedaleniguarda.it; 8 Department of Mechanical, Chemical and Materials Engineering, University of Cagliari, 09141 Cagliari, Italy; federico.arippa@unica.it; 9 Department of Surgical Sciences, University of Cagliari, 09124 Cagliari, Italy; marco.monticone@unica.it

**Keywords:** rehabilitation, disability, pain, self-assessment, exercise

## Abstract

Background. Evaluating the psychometric and clinical performances of the RM-18, the shorter version of the Roland Morris Disability Questionnaire (RMQ), in Italian people with non-specific low back pain (NSLBP) as a time-saving and clinically useful method of assessing disability. Methods. This cross-sectional study included 74 people (52 females and 22 males, 53.03 ± 15.25 years old) with NSLBP. The RM-18, the RMQ, the Oswestry Disability Index (ODI), and a pain intensity numerical rating scale (NRS) were administered. Psychometric testing included reliability by internal consistency (Cronbach’s alpha) and test–retest measurement (Intraclass Correlation Coefficient, ICC2.1), and concurrent validity by comparing the RM-18 with the RMQ and the ODI (Pearson’s r correlation). Two separate regression analyses were performed to investigate the different impact of RM-18 and RMQ on NRS. Results. Cronbach’s α of RM-18 was 0.92 and ICC (2,1) = 0.96. Strong correlations were found with the RMQ and the ODI (r = 0.98; r = 0.78, respectively). The regression models showed that the RM-18 and the RMQ similarly impacted the NRS (*p* < 0.001). Conclusion. The RM-18 showed satisfactory psychometric testing and similarly impacted the NRS when compared to the RMQ. It can be recommended for clinical and research purposes in Italian people with NSLBP.

## 1. Introduction

Non-specific low back pain (NSLBP) is a common symptom with an extremely variable progression, which hurts the lumbar spine, without a distinct, explicit, or anatomical cause such as spinal deformities, infections, inflammatory diseases, or tumors [1], and it is a worldwide health problem, affecting up to 80% of the adult population. To date, there is no “gold standard” or consensus treatment to alleviate symptoms and disability due to NSLBP, though the advocated interventions are numerous, with marked variations in costs and levels of supportive evidence. However, there is consensus that NSLBP management should be cost-effective, self-administered, educational, exercise-based, and use multi-modal and multi-disciplinary approaches [2].

Questionnaires for measuring pain and the functional status of individuals with Low Back Pain (LBP) focus on the activities of daily living and present responses for each question in a structured relationship between what the person can do/difficulties and pain: they are for the rehab specialist an important resource for measuring response to physiotherapy and progress over time.

In 1997, Paul W. Stratford and Jill M. Binkley published an article by pointing out that a modified version of the Roland Morris Disability Questionnaire (RMQ), a well-known self-reported tool that assesses activity limitations due to LBP, displayed equivalent reliability and concurrent validity when compared to the full version of the same instrument [3]. In more details, the original instructions, item phrasing, and scoring scheme were maintained in the modified version, which otherwise contained 18 out of the 24 original items [3]. The authors hence defined the shorter form of the RMQ as the RM-18 [3].

Patient-reported outcome measures (PROMs) like the RMQ are increasingly introduced in routine clinical practice when facing individuals with LBP, representing an aid to the evaluations of clinicians over time [4,5,6]. However, there is still difficulty to fully adopt PROMs like the RMQ regularly in the orthopedics and rehab fields [7,8]. Hence, alternative psychometrically sound versions of longer questionnaires like the RM-18 are welcomed to efficiently speed up the evaluations of spinal clinicians and remarkably spare the time spent with persons with LBP [9]. An example was recently brought to the attention of clinicians and researchers in the field of scoliosis [10].

An additional reason to adopt PROMs relies upon their tangible contribution to treatment choices and shared decision-making [11,12]. For instance, the possibility granted by an instrument like the RM-18 to collect valuable information on the physical limitations of people with LBP during common daily activities (ADLs) is noteworthy when building up rehabilitative programs based on functional exercises. In addition to general exercises involving spinal posture, mobility, or strengthening, these functional exercises are specifically targeted to transfers, walking, ascending stairs, and housework as those described in the RM-18 may favor recovery and prevent LBP recurrences [13]. Further, the expected decrease in spinal disability as a result of a PROM-driven rehabilitative program could also lead to improvement in pain perception: it is known that limitations during ADLs may nurture back ache in the course of time, as described by the fear–avoidance model of movement/(re)injury (FAM) which clearly relates disability to LBP [14].

Based on the premises above, the primary aim of this study was to evaluate the psychometric performance of the RM-18 in an Italian sample with non-specific LBP as a time-saving and clinically useful method of assessing disability. We conducted an assessment of reliability (internal consistency and test–retest reliability) and concurrent validity with standard measures of disability as originally proposed [3]. Additionally, the impact of the longer and the shorter versions of the RMQ on pain intensity perception was assessed in order to corroborate the wide body of literature on the FAM.

## 2. Materials and Methods

A Local Review Board endorsed this cross-sectional study (Protocol No. 24007; ERB DISPuTER, Department of Psychological Sciences, Health and Territory of G. D’Annunzio University of Chieti-Italy), which was carried out in accordance with the ethical and humane principles of research detailed in the Declaration of Helsinki [15].

This cross-sectional study involved persons consecutively (i.e., by a non-probability sampling technique) attending three Italian outpatient Hospital Rehabilitation Units, meeting the following inclusion criteria: diagnosis of non-specific LBP; people speaking Italian as their first language; and age over 18 years old. Exclusion criteria were specific causes of LBP (i.e., fracture, spinal deformity, disc herniation, canal stenosis, spondylolisthesis, or infections); peripheral or central neurological disorders assessed by case history and clinical evaluation; systemic illness; cognitive impairments; recent myocardial infarctions; any past cerebrovascular accidents; and refusal to give informed consent.

Individuals who satisfied the inclusion criteria were given further information about the study aims and procedures and requested to sign a written informed consent form. After that, demographic and clinical characteristics were collected, and all participants completed all the outcome measures reported below. Participants were asked to check the questionnaires if missing data were found by the staff; further, they were invited to fill in the RM-18 a second time, 7–10 days after their initial assessment, to avoid variations in symptoms linked to possible memory effects. No treatment was delivered in the interim period. The time to complete the RM-18 and the RMQ was gathered.

(1) RM-18. It is an 18-item PROM by which individuals are asked to value their capability of performing ADLs involving the spinal column. Six items from the original RMQ were deleted (i.e., nos. 2, 15, 17, 19, 20, 24) with the remaining scored 1 if the respondent considers its applicability to the specific action and 0 if not, with the total score ranging from 0 (no disability) to 18 (severe disability) [3].

(2) RMQ. It is a 24-item PROM by which people are requested to assess the ability of doing usual activities concerning their spine. Each item is scored 1 if the respondent considers its applicability to the specific action and 0 if not, with the total score ranging from 0 (no disability) to 24 (severe disability). The Italian validated version was used [16].

(3) Oswestry Disability Index (ODI). This PROM contains 10 items, which first section rates the intensity of LBP and the others its effects on daily activities. The score of each item ranges from 0 to 5, and the sum is expressed as a percentage of the maximum score, varying from 0 (no disability) to 50 (maximum disability). The Italian validated version was utilized [17].

(4) Pain intensity Numerical Rating Scale (NRS). An 11-point pain numerical rating scale ranging from 0 (no pain at all) to 10 (the worst imaginable pain) was used, asking participants to rate their current pain intensity [18].

All the questionnaires were self-administered. We decided to avoid the randomization of the order of the questionnaires to reduce the cognitive burden by respondents, and hence, the RM-18 was systematically distributed first, then the ODI, the NRS, and RMQ during the first assessment, respectively; only the RM-18-I was delivered during the second assessment.

### Statistics

Primarily, we tested the reliability and concurrent validity of the RM-18 as detailed below.

Reliability. Internal consistency was evaluated by calculating Cronbach’s alpha (values of >0.70 being considered acceptable); test–retest repeatability was examined in stable people using the intraclass correlation coefficient, ICC (2,1) (values of 0.70–0.85 were considered good and >0.85 excellent) [19].

Concurrent validity. Based on what was earlier assumed in a previous study on the same matter [3], it was hypothesized a priori that the RM-18 would achieve positive strong correlations with two standard measures of disability (RMQ and ODI, respectively). Pearson’s r correlation coefficients were calculated by considering low relationships if r ≤ 0.3, moderate if 0.3 < r < 0.5, and large if r ≥ 0.5 [19].

A sample of at least 50 people was needed to investigate reliability and concurrent validity as per the scope of the study [20].

Additionally, two separate hierarchical regressions were then performed to investigate the single ability of RM-18 and RMQ (independent variables) to impact pain intensity (NRS, dependent variable). Age and pain duration were considered as potential confounders and were consistently entered into the analyses to act as variates; considering three independent variates for multi-regressions, a sample size of at least 30 individuals was considered sufficient for the additional purpose of the study [21]. Level of significance was set at *p* < 0.05.

Statistics calculations were performed by means of IBM SPSS v.29 (Italian version).

The data associated with the paper are not openly accessible but are available from the corresponding author upon reasonable request.

## 3. Results

The study consecutively included 74 persons (52 females, mean age 53.03 ± 15.25 years) with non-specific LBP. Average pain duration was 96.86 ± 126.85 months. The socio-demographic characteristics of the individuals enrolled are shown in Table 1. The distribution of the RM-18 questionnaire in comparison with the other PROMs used in the study are shown in Table 2; there were no floor/ceiling effects.

The RM-18 took 57.24 ± 33.87 s (range 10–180) to complete. The questions were well received. No multiple responses were observed, nor were any comprehension difficulties raised during the instrument completion. The RMQ was completed in 67.80 ± 44.18 s (range 15–300).

Reliability. Cronbach’s α of RM-18 was 0.92 and test–retest reliability excellent: ICC (2,1) = 0.96 (95% CI: 0.93–0.97).

Concurrent validity. A priori hypotheses were all confirmed: RM-18 vs. RMQ r = 0.98; RM-18 vs. ODI r = 0.78.

**Figure 1:**
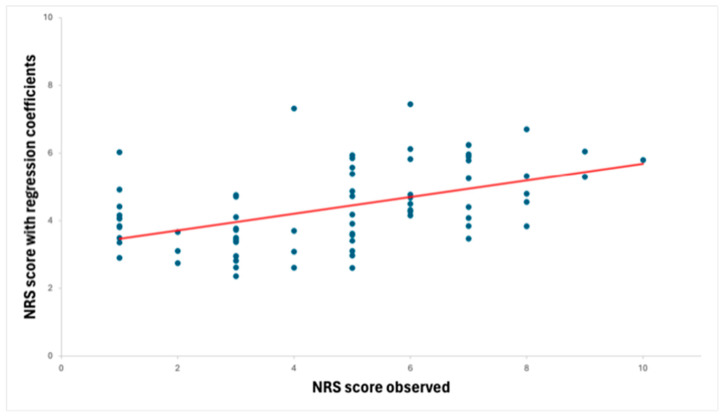
Scatter plot and regression curve of Predicted NRS versus Observed NRS. NRS = Numerical Rating Scale.

The regression models showed (Figure 1) that the RM-18 and the RMQ impacted the NRS similarly by respectively explaining the 32% of variance (F(3,70) = 10.99, *p* < 0.001) and the 32% of variance (F(3,70) = 10.76, *p* < 0.001). Individual contributions of each determinant of the NRS are reported in Table 3 and Table 4, respectively.

## 4. Discussion

The RM-18 showed adequate reliability and satisfactory concurrent validity in Italian-speaking people with non-specific LBP. Time of assessment of RM-18 was 15% shorter than RMQ. Moreover, the RM-18 demonstrated the same ability as the RMQ to impact pain intensity perception.

The internal consistency of the RM-18 was excellent and quite similar to that reported by the developers (0.91) [5], by showing a satisfactory degree of interrelatedness among items describing ADLs frequently affected by LBP.

Test–retest repeatability demonstrated an excellent level of agreement between the results on days 1 and 7–10, a value higher than that retrieved in the original study (0.86) [10]. The level of reliability noted in this investigation may reflect the fact that no treatment was provided to participants between the two testing occasions, a control not enacted in the original study [5].

As for concurrent validity, both correlations between RM-18 and the gold standard were achieved as pre-hypothesized. The highest correlation was with the RMQ given the similarity of most items tested. A strong correlation was also achieved when the ODI was considered, by confirming the correspondence in constructs of these PROMs. Similar findings were shown by the developers (0.99 with the RMQ and 0.82 with the ODI, respectively [5]).

The RM-18 and the RMQ impacted the NRS similarly, and this fact:

(i) does support the concrete possibility to adopt the shorter version when compared with the longer form: both PROMs were significant determinants of pain intensity, and hence, rehab professionals can safely also use the RM-18 during their clinical practice;
(ii) confirms the clockwise circularity of the FAM [14]: this finding should suggest that disability has to be considered as the primary reference point to personalize when people with non-specific LBP are addressed.

To the authors’ knowledge, a similar analysis was not previously conducted, and further replications of findings are hence recommended.

Clinical and rehabilitative implications of findings could also be pointed out:

(i) it is of importance to plan an adequate evaluation phase for the correct definition of individualized objectives for personal care [22,23]. It is known that each individual presents different characteristics from other people by pursuing short-, medium-, and long-term goals: by means of a correct evaluation, it is possible to precisely identify which objectives to work on [24,25].
(ii) people who feel accurately assessed and are helped to identify their own objectives will also be more compliant with the treatment with a notable saving of resources and time.
(iii) there will be the possibility of adapting the treatment to the person and not vice versa. For example, when an individual with non-specific LBP answers “yes” to question nos. 2 (I walk more slowly than usual because of my back), 8 (I get dressed more slowly than usual because of my back), 13 (I find it difficult to turn over in bed because of my back), or 16 (I avoid heavy jobs around the house because of my back) of the RM-18, the physiotherapist can gradually introduce and shape exercises within the rehab program, with the aim of training people during every session; videos on the management of specific ADLs should also be taken into account. Answering “no” to some issues should be regarded also as a resource that the person already has, by enhancing positive moods and self-efficacy and giving only brief hints while focusing more on the other troubling items.

### 4.1. Future Research and Prospective

The findings of this study may offer inspiration for future research. Indeed, it is worth continuing to analyze additional psychometric properties of the RM-18 in other clinical settings and cultures, in order to allow the possibility of systematic reviews of psychometric properties [20]. Updated information on content validity, appropriateness, acceptability, measurement error, and minimum detectable change are recommended [20]; further, the calculation of the minimal important clinical difference and predictive proficiency through prospective interventional studies is notable [20]. Moreover, it is of interest to capture possible relationships with patient-reported experience measures (PREMs) which gather individuals’ experiences of their healthcare and services [19]. The RM-18 was never investigated in people with learning problems, and this could be an additional research direction, such as replicating this study findings in separate temporal phases of people with LBP (i.e., acute, subacute, chronic). Finally, planning an adequately sized investigation based on a Structural Equation Model that includes the RM-18 and the other physical and psychological variables commonly included in the FAM would be innovative [14].

### 4.2. Possible Rehabilitative and Clinical Applications

The Italian validation of the Roland Morris—18 items can represent for the rehab specialist an important measurement tool that is short, repeatable, and easy to use by the patient with LBP, for the quantification of the result within the success of the therapy according to the Personalized Rehabilitation Plan. It is known that each individual presents different characteristics from other people by pursuing short-, medium-, and long-term goals: by means of a correct evaluation, it is possible to precisely identify which objectives to work on. Furthermore, people who feel accurately assessed and are helped to identify their own objectives will also be more compliant with the treatment with a notable saving of resources and time. Hopefully, there will be the possibility of adapting the treatment to the person and not vice versa.

### 4.3. Limits

This study should acknowledge some limitations. First, the study design is cross-sectional; thus, responsiveness and minimal important change could not be assessed. Second, the association between back disability and physical performance measures was not investigated as only questionnaires were employed. Third, relationships with other psychological characteristics (e.g., Pain Self-Efficacy Questionnaire or the Coping Strategies Questionnaire-27 revised) [26,27], or quality of life (e.g., the Short-Form Health Survey 36-items) [28], were not examined. Fourth, our research was limited to people with non-specific LBP. Whether these results can be expanded to individuals with other causes of lumbar pain (e.g., canal stenosis, fracture, or disk herniation) should be further verified. Fifth, the study was conducted in an Italian sample. Sixth, given the cross-sectional feature of the research, regression weights should not be confounded with causal association.

## 5. Conclusions

Our results show that the evaluation process of ADLs in people with non-specific LBP can be shortened by also using the RM-18 rather than the longer RMQ, without the risk of losing its relevance. The RM-18 showed satisfactory reliability and concurrent validity and similarly impacted pain intensity when compared to the RMQ. The RM-18 can be hence recommended for use in clinical and research settings for the assessment of Italian-speaking people with non-specific LBP.

## Figures and Tables

**Table 1:** Socio-demographic characteristics of the study population (n = 74).

Age (years), mean ± SD		53.03 ± 15.25
Gender (n)	Male	22 (29.7%)
	Female	52 (70.3%)
Marital status (n)	Married	47 (63.5%)
	Single	17 (23%)
	Divorced	5 (6.8%)
	Widowed	5 (6.8%)
Employment (n)	Students	4 (5.4%)
	Employed	43 (58.1%)
	Self-employed	6 (8.1%)
	Domestic works	3 (4.1%)
	Retired	17 (23%)
	Missing	1 (1.4%)
Education level (n)	Primary school	5 (6.8%)
	Middle school	7 (9.5%)
	High school	32 (43.2%)
	University	30 (40.5%)
Smokers (n)	Yes	19 (25.7%)
	No	55 (74.3%)
Alcohol (n)	Yes	20 (27%)
	No	54 (73%)
Physical activity (n)	Yes	40 (54.1%)
	No	34 (45.9%)
Comorbidities (n)	None	53 (71.6%)
	Cardiac	8 (10.8%)
	Respiratory	4 (5.4%)
	Gastrointestinal	4 (5.4%)
	Renal	1 (1.4%)
	Headache	4 (5.4%)
Body Mass Index (kg/m^2^), mean ± SD		25.14 ± 3.86

SD: standard deviation; n: raw number; %: percentage.

**Table 2:** Distribution of RM-18 questionnaire, RMQ, ODI, and pain intensity NRS scores.

	Mean	SD	25th%	50th%	75th%	Floor Effect [%]	Ceiling Effect [%]
RM-18 (0–18)	7.31	5.64	2	6	13	0	0
RMQ (0–24)	8.47	6.53	3	7	14.25	0	0
ODI (0–50)	12.14	9.41	4.75	9	17	0	0
NRS (0–10)	4.59	2.36	3	5	6.25	0	0

RM-18: Roland Morris—18 items; RMQ: Roland Morris Disability Questionnaire; ODI: Oswestry Disability Index; NRS: numerical rating scale.

**Table 3:** Hierarchical regression coefficients for the model with NRS as outcome and RM-18, age, and pain duration as determinants.

Outcome	Determinants	Coefficient	95% CI	t	*p* Value
NRS	RM-18	0.162	0.061–0.263	3.209	0.002
	Age	0.045	0.010–0.081	2.524	0.014
	Pain Duration	−0.001	−0.005–0.003	−0.397	0.692
	Constant	1.091	−0.624–2.806	−0.269	0.209

NRS = Numerical Rating Scale; RM-18 = Roland Morris—18 items.

**Table 4:** Hierarchical regression coefficients for the model with NRS as outcome and RMQ, age, and pain duration as determinants.

Outcome	Determinants	Coefficient	95% CI	t	*p* Value
NRS	RMQ	0.139	0.050–0.228	3.125	0.003
	Age	0.047	0.011–0.083	2.620	0.011
	Pain Duration	−0.001	−0.006–0.003	2.620	0.596
	Constant	1.043	−0.676–2.762	1.210	0.230

NRS = Numerical Rating Scale; RMQ = Roland Morris Disability Questionnaire.

## Data Availability

The data supporting the findings of this study are available upon request from the corresponding author.
